# ArnR binds a [4Fe–4S] cluster and indirectly senses anaerobicity in *Corynebacteria*

**DOI:** 10.1093/mtomcs/mfaf026

**Published:** 2025-07-21

**Authors:** Jason C Crack, Lauren R Harvey, Katie E Johnson, Nick E Le Brun

**Affiliations:** From the Centre for Molecular and Structural Biochemistry, School of Chemistry, Pharmacy and Pharmacology, University of East Anglia, Norwich, United Kingdom; From the Centre for Molecular and Structural Biochemistry, School of Chemistry, Pharmacy and Pharmacology, University of East Anglia, Norwich, United Kingdom; From the Centre for Molecular and Structural Biochemistry, School of Chemistry, Pharmacy and Pharmacology, University of East Anglia, Norwich, United Kingdom; From the Centre for Molecular and Structural Biochemistry, School of Chemistry, Pharmacy and Pharmacology, University of East Anglia, Norwich, United Kingdom

## Abstract

*Corynebacteria* are commercially and medically important Gram-positive bacteria that can switch from aerobic to anaerobic respiration in response to low O_2_ and the availability of nitrate as an alternative electron acceptor. The *narKGHJI* operon encoding the respiratory nitrate reductase is under the control of a novel regulator, ArnR, which plays a major role in the aerobic/anaerobic respiratory switch. ArnR was previously shown to be an iron–sulfur cluster protein that modulates its DNA binding according to availability of O_2_. However, previous data suggest that it does not do this directly in response to O_2_, but instead by sensing nitric oxide (NO), which builds up only under low O_2_ through the activity of nitrate reductase. Here, we report spectroscopic and mass spectrometric studies of *C. glutamicum* ArnR and its reactions with O_2_ and NO. We demonstrate that ArnR is a dimer that binds a [4Fe–4S] cluster in each subunit, and this form of the protein binds tightly to DNA. The [4Fe–4S] cluster of AnrR degrades only very slowly in the presence of O_2_, consistent with the ability of ArnR to repress *nar* transcription under aerobic conditions. Reaction with NO results in the formation of mono- and di-nitrosylated forms of the [4Fe–4S] ArnR dimer, which exhibit altered DNA-binding characteristics such that the di-nitrosyl form no longer binds to promoter DNA (i.e. cluster degradation is not required in order to modulate DNA binding). These data are consistent with previous literature and lead us to propose a model for AnrR regulatory function.

## Graphical Abstract

**Graphical Abstract:**
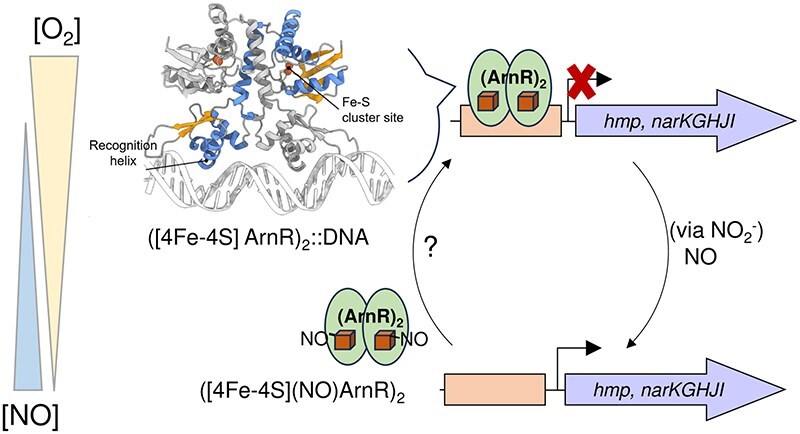

## Introduction

*Corynebacteria*  are a genus of Gram-positive bacteria that are widely distributed in nature, with representatives being part of the normal human microbiota [1]. The role of *Corynebacteria* in the development of disease is well known (e.g. *Corynebacterium diphtheriae* [2]) but other, lesser-known *Coyrnebacteria sp*. are now increasingly being recognized as opportunistic human pathogens (e.g. *C. ulcerans*), or the source of potent carcinogens in contaminated smokeless tobacco products (e.g. *C. ammoniagenes*) [3–6]. *Corynebacteria* are also commercially important, with *C. glutamicum* being widely used for the industrial production of l-glutamic acid and l-lysine. Other amino acids produced by *C. glutamicum*, including l-aspartic acid, l-threonine, l-alanine, l-valine, and l-isoleucine, are used as building blocks for the pharmaceutical industry or serve as nutritional supplements [7, 8].

O_2_ limitation is a common problem in the efficient production of amino acids by *C. glutamicum*, leading to reduced yields and accumulation of unwanted organic acids [9]. Many *Corynebacteria*, including *C. diphtheriae* and *C. glutamicum*, are facultative anaerobes capable of utilizing nitrate (NO_3_^−^) as a terminal electron acceptor to support growth in the absence of O_2_, potentially enhancing l-lysine and l-arginine production [9–11]. *Corynebacterium glutamicum* was found to consume extracellular nitrate and to excrete nitrite (NO_2_^−^) during anaerobic conditions, but not aerobic conditions [11]. The utilization of nitrate was attributed to the presence of a *narKGHJI* operon, which encodes a membrane-associated nitrate reductase (NarGHI) that reduces nitrate (NO_3_^−^) to NO_2_^−^ and helps maintain the proton motive force, facilitating growth. The operon also encodes a transporter (NarK) to supply nitrate reductase with substrate (NO_3_^−^) and excrete excess product (NO_2_^−^), together with a chaperone (NarJ) that participates in the maturation of the nitrate reductase enzyme [9, 11–13].

Consistent with the *Escherichia coli* paradigm, *C. glutamicum narKGHJI* nitrate reductase expression is repressed during aerobic growth, but activated under anaerobic conditions in the presence of nitrate [11, 12]. In *E. coli*, the *narGHJI* operon is regulated by the global fumarate and nitrate reduction (FNR) transcriptional regulator [14], which upon acquisition of an O_2_-sensitive [4Fe–4S] cluster activates *narGHJI* expression under anaerobic conditions. *Corynebacterium glutamicum* lacks a direct FNR homologue [15–17] and so must regulate nitrate respiration in a different way. Nishiumura *et al*. found that a gene located immediately downstream of the *narKGHJI* operon encoded a putative transcriptional regulator, ArnR, and demonstrated its importance for the repression of nitrate reductase under aerobic conditions [11, 12, 18]. Subsequent work identified *C. glutamicum* GlxR (a member of the CRP/FNR super family) as a cyclic-AMP (cAMP)-dependent activator of *narKGHJI* expression in response to energy metabolism [19].

Anaerobic nitrate respiratory growth is potentially hazardous: nitrite, the initial product of nitrate reduction, is potentially toxic if allowed to accumulate [20]. Further reduction of nitrite by nitrate reductase (or other proteins) results in the production of the cytotoxic radical nitric oxide (NO), a contributory factor in nitrosative stress [21–24]. Many bacteria experience nitrosative stress when NO, or other reactive nitrogen oxides derived from NO chemistry, impair the function of cellular components [22, 25]. To mitigate the deleterious effects of NO, most bacteria mount a complex and multifaceted response that is coordinated by NO-sensitive transcriptional regulators [26–28]. In many cases, the NO-sensitive response regulator NsrR fulfills this role [29].

NsrR belongs to the iron–sulfur (Fe–S) cluster-containing clade of the Rrf2 superfamily of transcriptional regulators [29]. Fe–S cluster-NsrR represses transcription in the absence of NO, with the *hmp* gene the principal target of repression in multiple species [26, 30–33]. The encoded Hmp protein is a flavohaemoglobin oxygenase that rapidly converts NO to NO_3_^−^ under (micro)aerobic conditions [34, 35]. In *C. glutamicum*, which does not contain a direct NsrR homologue, ArnR is also responsible for the repression of the *hmp* gene [18, 28].

ArnR contains three conserved cysteines (Cys179, 193, and 223), and does not share significant sequence homology with any known O_2_/NO-sensitive transcriptional regulators (CRP/FNR, Rrf2, or WhiB-like). However, it does contain a winged helix-turn-helix (wHTH) DNA-binding domain reminiscent of MarR/AsrR transcriptional regulators (e.g [4Fe–4S] SufR) [12, 18, 36–40]. Intriguingly, as-isolated ArnR was found to bind an Fe–S cluster of an unknown type. Nishiumura *et al* demonstrated specific binding of Fe–S ArnR to the *narK* and *hmp* promoter fragments, and showed that NO, but not NO_2_^−^, eliminated DNA binding [12, 18]. Single cysteine variants (Cys to Ala) failed to acquire an Fe–S cluster or to bind to *narK* or *hmp* promoter fragments [12, 18], indicating the importance of the cluster for regulatory function.

Here, we report investigations of the biochemical properties of ArnR. Using a combination of optical (UV–vis absorbance and circular dichroism) spectroscopy and electrospray ionization-mass spectrometry (ESI-MS), together with surface plasmon resonance (SPR), we present information on the nature of the Fe–S cluster, its reaction with O_2_ and NO, as well as the effect these have on DNA binding. We compare our findings to those previously reported for ArnR and other NO-sensitive transcriptional regulators, and propose a model for ArnR function.

## Materials and methods

### Purification of *C. glutamicum* ArnR

Plasmid pArnR, based on pET21a and encoding a C-terminal His-tagged variant of ArnR (Cgl1185) from *C. glutamicum* ATCC 13032, was purchased from Genscript. ArnR was overproduced in aerobically grown *E. coli* strain BL21λDE3 transformed with pArnR, as previously described [12, 41]. All steps were carried out anaerobically unless otherwise stated. Cell pellets were resuspended in buffer A [50 mM tris(hydroxymethyl)aminoethane (Tris) -HCl, 150 mM NaCl, 5% (v/v) glycerol, pH 8], removed from the anaerobic cabinet, sonicated on ice, and returned to the anaerobic cabinet. The cell suspension was transferred to O-ring sealed centrifuge tubes (Nalgene) and centrifuged outside of the cabinet at 40 000 × *g* for 45 min at 1°C. The supernatant was loaded onto a HisTrap column (2 × 5 ml; Cytiva) and washed with lysis buffer containing 5% eluting buffer B (buffer A, with 500 mM imidazole) until *A*_280 nm_ ≤ 0.1. Bound proteins were eluted (2 ml/min) using a linear gradient (15 ml) from 5% to 100% buffer B. Fractions (1 ml) containing ArnrR were pooled and loaded onto a HiTrap heparin column (5 ml; Cytiva), washed with buffer A and manually eluted using buffer C (buffer A, with 2 M NaCl). Coloured fractions containing ArnR were pooled and stored in an anaerobic freezer (−35°C, mBraun) until needed. Where necessary, gel filtration was carried out using a Sepharcyl S-100HR 16/50 column (Cytiva) equilibrated with buffer A with a flow rate of 1 ml/min. Protein purity was judged by sodium dodecyl sulfate-polyacrylamide gel electrophoresis (SDS-PAGE) and routine liquid chromatography-mass spectrometry (LC-MS). Apo ArnR was prepared from holo protein by aerobic overnight incubation with 5 mM ethylenediaminetetraacetate (EDTA) followed by desalting.

### Protein, metal, and sulfide determinations

ArnR concentrations were determined using the methods of Smith (Pierce) with bovine serum albumin as the standard. Iron content was determined using Ferene, as previously described [42]. Acid-labile sulfide was determined according to the method of Beinert [43]. Zinc content was determined using a commercially available kit (product 17255, Sentinel Diagnostics, Italy), according to the manufacturer’s instructions, except all volumes were doubled for use with standard 1 ml cuvettes (1 cm pathlength). Here, zinc is chelated by (2-5-bromo-2-pyridylazo)-5-(N-propyl-N-sulfo-propylamino) phenol (5-Br-PAPS) and detected at *A*_570 nm_, while masking agents eliminate interference from iron and copper ions [44].

ArnR samples were also analysed by inductively coupled plasma–mass spectrometry (ICP-MS). Briefly, as isolated or apo ArnR was diluted to 0.5 ml (20—100 µM final concentration), treated with 0.2 ml ultrapure hydrogen peroxide (Fisher Trace element grade), then 0.2 ml ultrapure nitric acid (Fisher Chemical Optima grade), and digested overnight. The sample was diluted to 4.5 ml with ultrapure Milli-Q water and treated with 0.5 ml of ^103^Rh internal standard (Alfa Aesar Rhodium standard solution 100 µg/kg). Samples were then infused into the source of an iCAP-TQ ICP-MS spectrometer (Thermo Fisher) and analysed for a range of transition metals and sulfur. ArnR contains several sulfur-containing amino acids (3 Cys, 6 Met), with additional Fe and S being derived from the iron–sulfur cluster. For ICP-MS, molar ratios of iron and zinc (and other metal ions) per polypeptide were determined from sulfur determinations corrected for cluster-derived sulfur by assuming total iron was equal to total cluster sulfur.

### Electrophoretic mobility shift assays

Labelled (5’ 6-FAM) self-complementary, single stranded oligonucleotides (80 bp) carrying the *C. glutamicum hmp* promoter sequence were purchased from Eurofins (Eurofins Genomics) and dissolved in DNAse-free water to give 200 µM stock solutions (Table S1). Band shift reactions (20 µl) were carried out in 10 mM Tris 54 mM KCl, 0.3% (v/v) glycerol, pH 7.5, as previously described [30]. Briefly, 1 µl of DNA was titrated with aliquots of ArnR (20 µl final volume), typically to a 30-fold molar excess, and incubated on ice for ∼2 min. Loading dye (2 µl, containing 0.03% (w/v) bromophenol blue) was added and the reaction mixtures were immediately separated at 30 mA for 30 min on a 5% (w/v) polyacrylamide gel in 1 × TBE (89 mM Tris, 89 mM boric acid, and 2 mM EDTA), using a Mini Protean III system (BioRad). Gels were visualized (Ex_473 nm_) on a GE Typhoon FLA 9000 Scanner (Cytiva). Polyacrylamide gels were pre-run at 30 mA for 2 min prior to use.

### Spectroscopic experiments

UV–visible absorbance and circular dichroism (CD) were recorded using a Jasco V550 spectrometer and a Jasco J810 spectropolarimeter, respectively. Samples were prepared and manipulated in an anaerobic glovebox (O_2_ <10 ppm) and measured in 1 cm pathlength anaerobic quartz cuvettes. For O_2_ experiments, protein samples were rapidly diluted with aerobic buffer A to give the desired O_2_ concentration, and the cluster response immediately followed by spectroscopy. For NO experiments, ArnR was diluted into buffer D [100 mM 4-(2-hydroxyethyl)piperazine-1-ethanesulfonic acid (HEPES), 500 mM NaCl, 5% glycerol, pH 7.5] and titrated with varying aliquots of proline NONOate (4 mM NONOate, in 50 mM NaOH; Cayman Chemicals) to achieve the desired [NO]: [4Fe–4S] ratio before spectra were recorded.

### Surface plasmon resonance

The ReDCaT principle of SPR was used to study the interaction of ArnR with the *hmp* promoter. Briefly, double stranded DNA oligonucleotides (100 µM dsDNA), were annealed from equimolar concentrations of single stranded oligonucleotides (Table S1) by heating to 70°C for 10 min, followed by cooling. ReDCaT oligos were diluted to 100 µM and annealed to give 50 µM dsDNA. Biotinylated dsDNA ReDCaT oligos were diluted further to ∼1 nM with 0.01 M HEPES pH 7.4, 0.15 M NaCl, 0.005% v/v Surfactant P20 (HBS + P) buffer and captured on a strepavadin sensor chip (SAD200L, Xantec Bioanalytics GmbH) to a density of ∼60 RU for all flow cells. Next, complementary ReDCaT oligos were dissociated from working flow cells using regeneration buffer (50 mM NaOH, 1 M NaCl), and replaced with the dsDNA *hmp* promoter sequence containing ReDCaT linker sequence on the complementary strand, as previously described [45, 46].

Prior to use, an aliquot (260 µl) of ArnR was exchanged into 0.1 M HEPES, 1.5 M NaCl, and 0.5% polysorbate 20, pH 7.4 via Zeba spin desalting columns [∼7 kDa molecular weight cut off (MWCO), Thermo Scientific] and the volume increased to 1 ml with 10 mM HEPES, 150 mM NaCl, and 0.05% polysorbate 20, pH 7.4 (HBS + P) to give a stock solution of >90 µM [4Fe–4S]. For SPR analysis, ArnR was diluted a final concentration of 0–1125 nM [4Fe–4S] with HBS + P with 1 mM dithiothreitol (DTT) and injected in parallel over the immobilized dsDNA surfaces, with association and dissociation phase of 130 and 150 s, respectively. Between runs, chip surfaces were washed with 2 M NaCl in HBS + P to disrupt protein-DNA interactions. All SPR measurements were performed at 25°C on a Biacore S200 (Cytiva) using a multi cycle approach, with anaerobic HBS + P with 1 mM DTT as the running buffer. Sensorgrams were recorded with a 40 Hz data rate from each flow cell and referenced against a channel containing immobilized ReDCaT dsDNA.

SPR sensorgrams were initially processed with Biacore evaluation software (Cytiva). The analyte response for each flow cell was normalized to the maximum observed analyte response, then averaged, as previously described [45]. The relative response was then plotted against the concentration of [4Fe–4S] ArnR and fitted to a simple binding equation (see Equation 1), where *Y_max_* is the maximum relative binding response, *x* is the concentration of [4Fe–4S] ArnR, and *K_d_* is the binding affinity. Fitting was performed using Origin Pro 2024 SR1 (OriginLab Corp.):


(1) \begin{eqnarray*}
\textit{Relative}\ \textit{Response} = ({{Y}_{max}}x)/({{K}_d} + x).
\end{eqnarray*}

### Preparation of oligonucleotides for ESI-MS

High-purity, salt-free, self-complementary, single-stranded oligonucleotides were purchased from Eurofins (Eurofins Genomics) and dissolved in DNAse-free water to give 200 µM stock solutions. Double-stranded DNA oligonucleotides (dsDNA), containing the ArnR promoter sequence from *C. glutamicum hmp* (Table S1) were annealed from equimolar concentrations of single stranded oligonucleotides by heating to 70°C for 10 min. After cooling, dsDNA was exchanged into 100 mM ammonium acetate, pH 8, via Zeba spin desalting columns (∼7 kDa MWCO, Thermo Scientific). The dsDNA content was determined using the sum of the extinction coefficients (ε_260 nm_, Novopro biosciences Inc., China) for the appropriate single stranded oligonucleotides.

### Mass spectrometry measurements

ArnR was buffer-exchanged into 250 mM ammonium acetate, pH 8.0, under anaerobic conditions using PD MiniTrap G-25 desalting columns (Cytiva) to give stock solution of ∼100 µM cluster. For ESI-MS of protein-DNA complexes, the protein was exchanged into 100 mM ammonium acetate, pH 8.0. Prior to ESI-MS analysis, aliquots of the protein stock solution were diluted to ∼8 µM cluster with the appropriate ammonium acetate solution and combined with an aliquot of promoter DNA, as required (200 µl, final volume).

Protein samples were infused directly (5 µl/min) into the source of a Bruker micrOTOF-QIII mass spectrometer (Bruker Daltonics, Coventry, UK) operating in the positive ion mode, and calibrated using Agilent ESI-L low concentration tune mixture (Agilent Technologies). MS data were continuously acquired over the *m/z* range 1000–6000 for 5 min using Bruker oTOF control software, with parameters as follows: dry gas flow 4 l/min, nebulizer gas pressure 0.8 bar, dry gas 180°C, capillary voltage 3.5 kV, offset 0.5 kV, ion energy 5 eV, collision RF 1500 V_pp_, and collision cell energy 10 eV. Processing and analysis of MS experimental data were carried out using Compass Data Analysis version 4.1 (Bruker). Neutral mass spectra were generated using the ESI Compass version 1.3 Maximum entropy deconvolution algorithm over a mass range of 20–30 kDa for the monomer, 50–60 kDa for the dimer, and 52–72 kDa for ArnR-DNA complexes. Exact masses are reported from the peak centroids representing the isotope average neutral mass. For apo proteins, masses were derived from *m/z* spectra, where peaks corresponded to $[ {M + zH} ]/z$, where *M* is the molecular mass of the protein, *H* is the mass of the proton, and *z* is the charge of the ion. For holo proteins, where the change of the cluster contributes to the overall charge of the ion, the peaks correspond to $[ {M + [ {FeS} ] + ( {z - x} )H} ]/z$, where [*FeS*] is the mass of the Fe–S cluster, the other variables are the same as above. Here, the charge of the Fe–S cluster, represented by *x*, offsets the number of protons required to achieve an ion of *z* charge. Post deconvolution, the observed mass is typically off set from the predicted mass by the contributing charge, *x*, according to$[ {M + ( {[ {FeS} ] - x} )} ]$ [47].

## Results

### Homology modelling of ArnR

*Corynebacterium glutamicum* ArnR (Cgl1185) shares no significant sequence homology with NO-sensing members of the Rrf2, Wbl, or CRP/FNR family transcriptional regulators. Homologues of ArnR are found almost exclusively in Actinobacteria, with ∼30% of *Corynebacterium sp*. containing ArnR, nitrate reductase (*narKGHJI*) and flavohaemoglobin oxygenase (*hmp*) [6]. As a high-resolution structure of ArnR is not yet available, the ArnR sequence was submitted to Interpro [38]. This indicated the presence of a N-terminal wHTH DNA-binding domain, similar to those of the MarR/ArsR family of transcriptional regulators, as previously noted [12, 39].

**Figure 1.**
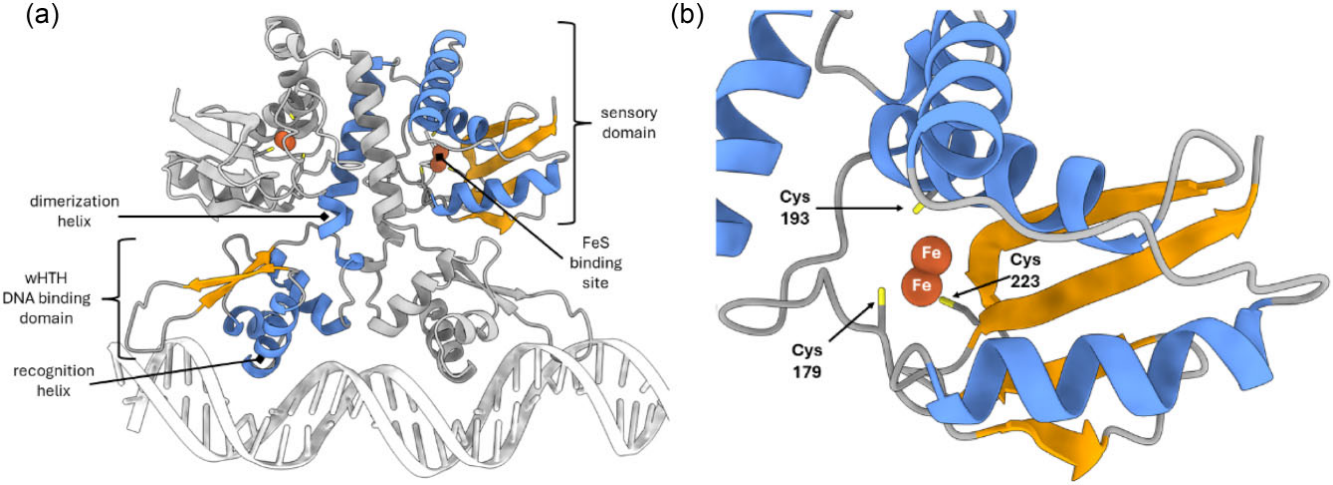
Alphafold3 model for ArnR-DNA complex. (a) Model obtained upon submission of the ArnR amino acid sequence, the *hmp* promoter sequence and Fe ions to the alphafold3 server. Predicted domains include an N-terminal wHTH, and putative C-terminal sensory domain, separated by a dimerization helix (labelled for one of the two protomers). (b) Close up view of the putative sensory domain showing the predicted location of two Fe ions and three conserved Cys residues that are likely to be ligands to the Fe–S cluster [18].

No significant sequence homology was identified for the C-terminal portion of ArnR. Therefore, the ArnR amino acid sequence, the *hmp* promoter sequence (JCC04/05, Table S1) and Fe ions were submitted to the Alphafold3 server [48]. The resulting Alphafold 3 model featured the wHTH domain, which is separated from a putative C-terminal sensory domain by a dimerization helix (Fig. 1a). Alphafold 3 placed two Fe ions within the putative C-terminal sensory domain, in close proximity to the three conserved cysteine residues (Cys179, 193, and 223) that were identified by Nishiumura *et al*. as likely ligands to an Fe–S cluster [18, 48] (Fig. 1b). Generally, Fe–S cluster are anchored to the protein framework by four ligands irrespective of cluster type. In most cases cysteine residues are the preferred ligand, but aspartate, glutamate, histidine, or serine residues can substitute for one or more of the cysteine residues [45, 49, 50]. Here, there is no fourth conserved Cys residue. Sequence alignment of ArnR homologs indicates a conserved His residue in the vicinity of the cluster (Fig. S1), but experimental evidence in support of this or any other potential ligand is currently missing.

To learn more about the putative sensory domain, the corresponding region of the Alphafold3 model was submitted to the Fold Seek server [51], which aligns the structure of the query molecule against structures from multiple databases, including the AlphaFoldDB [52], ESM-Atlas [53], CATH50 [54], and the protein data bank (PDB) [55]. Fold Seek indicated that weak sequence homology (∼20% identity) exists between the putative ArnR sensory domain and the C-terminal portion of Uniprot I6Y187 (Rv2621c) from *Mycobacterium tuberculosis*. Rv2621c appears to be an ArnR-like transcriptional regulator with weak sequence homology to the diverse TRAPPC3 family of trafficking protein particle complexes (CATH superfamily 3.30.1380.20) through the putative C-terminal sensory domain. Swiss Model also identified the zinc bound form of TRAPPC3 (PDB: 7YH2, 7YH3) as a potential template (∼14% identity) for sequence homology modelling of the putative sensory domain of ArnR [56]. We note that the coordinating ligands utilized by Zn^2+^ ions may often resemble those utilized by Fe–S proteins [57] (Fig. S2).

### Anaerobic purification of ArnR results in a [4Fe–4S] cluster-bound form

**Figure 2.**
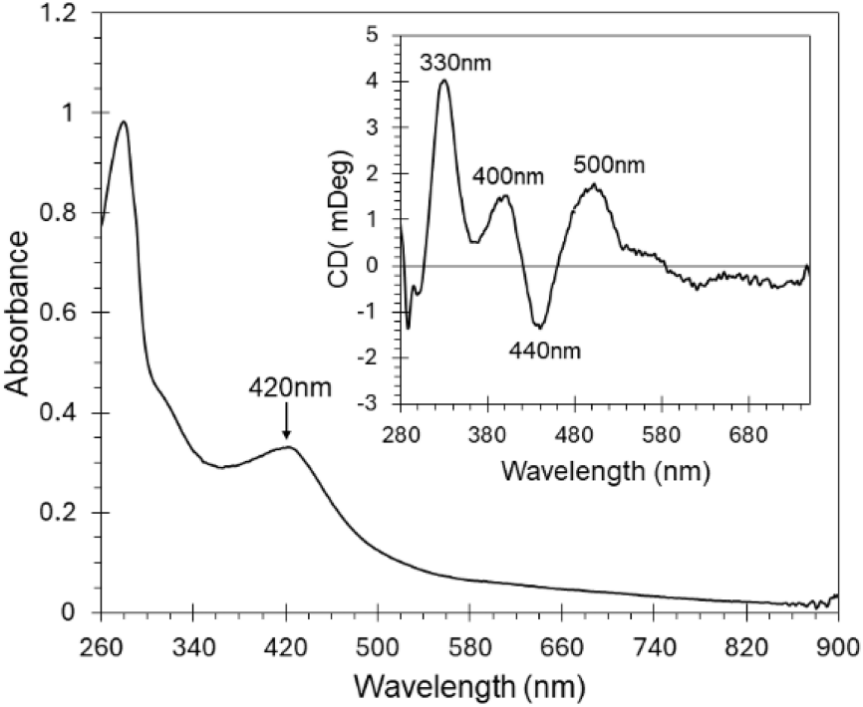
Optical spectroscopy of as-isolated ArnR. The absorption spectrum of as isolated ArnR displays features characteristic of a [4Fe–4S] cluster, with a ɛ_420 nm_ of ∼13.5 ± 0.5 mM^−1^ cm^−1^. Inset shows the unique CD band pattern of the ArnR cluster.

It was previously reported that ArnR preparations were coloured with absorbance properties consistent with the presence of an Fe–S cluster of an unknown type [12, 18]. To learn more about the protein and associated cluster, we purified ArnR under strictly anaerobic conditions following overproduction in *E. coli*. Anaerobic solutions of as-isolated ArnR were golden brown with a broad UV–visible absorption spectrum and a prominent peak at 420 nm, consistent with the presence of a [4Fe–4S] cluster (Fig. 2) [58]. Because the electronic transitions of the Fe–S cluster gain optical activity from the asymmetric fold of the protein in which they are bound, the CD band pattern reflects the local cluster environment. The anaerobic CD spectrum of ArnR displayed positive (+) features at 330, 400, and 500 nm and a negative feature (−) at 440 nm (Fig. 2, inset).

As isolated, ArnR was found to contain 3.8 ± 0.2 Fe, 4.1 ± 0.3 S, and 0.6 ± 0.1 Zn ions per protein by colorimetric methods. ICP-MS indicated 4.2 ± 0.9 Fe and 0.4 ± 0.1 Zn ions per protein, consistent with colorimetric methods, and also that ArnR did not contain significant levels of any other metal ions. Taken together, the data indicated that as isolated, ArnR was fully loaded with [4Fe–4S] clusters. Apo ArnR contained 0.15 ± 0.05 Fe ions per protein, as determined by ICP-MS and colorimetric assay. Apo ArnR was also found to contain ∼0.5 Zn per protein (i.e. similar levels to those of as-isolated AnrR), indicating either that bound Zn is resistant to/protected from chelator treatment, or that an unknown source of Zn contamination similarly affects both as-isolated and apo AnrR. The physiological relevance, if any, of ArnR-bound Zn is unclear and beyond the scope of the current work. However, we note that some iron–sulfur proteins feature Zn-binding sites [59, 60].

Previously, substitution of each of the three conserved cysteine residues (Cys179, 193, and 223) of ArnR, located towards the C-terminus of the protein, by Ala resulted in loss of the Fe–S cluster and loss of DNA binding [18]. The lack of a fourth cysteine ligand may also be reflected in the extinction coefficient at 420 nm due to the Fe–S cluster, which was determined to be ∼13.50 ± 0.5 mM^−1^ cm^−1^, comparable to other transcriptional regulators containing [4Fe–4S](Cys)_3_(X) clusters [30, 45, 50].

**Figure 3.**
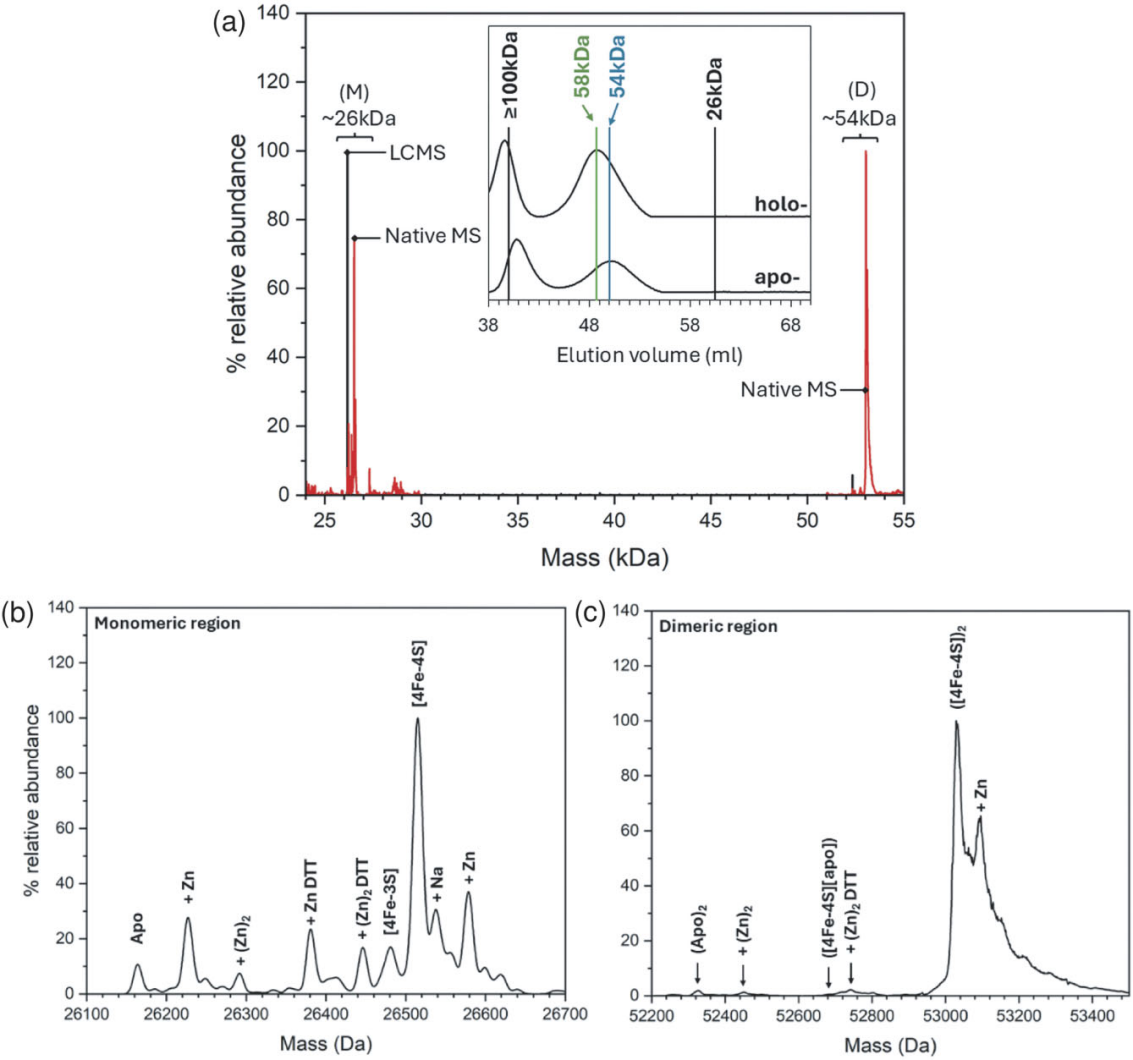
Native MS of ArnR. (a) Wide range deconvoluted LC-MS and native MS spectra of ArnR (as labelled) indicate the presence of monomeric (M) and dimeric (D) species. Inset, gel filtration chromatogram of ArnR, confirming that ArnR is a dimer in solution, irrespective of the cluster. Deconvoluted monomeric (b) and dimeric (c) regions are consistent with the presence of [4Fe–4S] clusters during native MS.

To determine the association state of ArnR, analytical gel filtration was performed (Fig. 3, inset). In the absence of O_2_, the majority of [4Fe–4S] ArnR eluted with a relative molecular mass of ∼58 kDa. Aerobically generated apo ArnR eluted with mass of ∼54 kDa, indicating ArnR is a constitutive homodimer in solution, irrespective of the status of the cluster. Gel filtration also revealed the presence of a larger ArnR species eluting within the void volume of the column, indicating a mass ≥100 kDa.

As Fe–S clusters are acid labile, they are lost under the mildly acidic and denaturing conditions of LC-MS (Fig. 3a, black line). An alternative approach, termed native MS, can be used to preserve the protein-structure in the gas phase, enabling non-covalent protein-cofactor and protein–protein interactions to be studied [47, 61, 62]. The deconvoluted native mass spectrum of ArnR contained two principal peaks (Fig. 3a, red line) that were not present in the LC-MS spectrum. The first was centred on 26.5 kDa, close to the monomeric mass, while the second was centred on 53.2 kDa, close to the dimeric mass of ArnR. A further species due to a tetrameric form of ArnR was also observed (Fig. S3), which may correspond to the high mass species observed by analytical gel filtration.

The partial dissociation of dimeric proteins into monomers during the transition to the gas phase is a well-known phenomenon [63]. In the case of Fe–S cluster proteins, this can prove advantageous because it simplifies the assignment of Fe–S species [16, 64]. The monomer region of the spectrum contained several peaks. The dominant peak at 26 515 Da corresponded to an ArnR monomer carrying a [4Fe–4S]^2+^ cluster (predicted mass, 26 516 Da), while adduct peaks to the higher mass side resulted from the presence of Na^+^ ions. Apo protein was observed at 26 166 Da via LC-MS (predicted mass, 26 166 Da), but was observed at 26 164 Da by native MS, indicating the likely presence of a disulfide bond; apo protein adducts at + 64 and +216 Da likely correspond to contaminating Zn^2+^ and Zn^2+^-DTT, respectively (Fig. 3b).

In the dimer region of the spectrum, the dominant peak was at 53 032 Da, corresponding to the ArnR dimer containing two [4Fe–4S]^2+^ clusters (predicted mass 50 032 Da). To the high mass side was a prominent +63 Da adduct (likely due to Zn^2+^), together with series of poorly resolved Na^+^ adducts. To the low mass side were smaller peaks from ArnR dimers containing one [4Fe–4S] cluster (hemi-apo), or no cluster (apo) (Fig. 3c). Thus, in solution ArnR primarily exists as homodimer with one [4Fe–4S] cluster per subunit (see Table 1 for a comparison between observed and predicted masses).

**Table 1.** Predicted and observed masses for selected ArnR species.

Species	Predicted mass^a^ (Da)	Observed mass^b^ (Da)	ΔMass^c^ (Da)
**ArnR (monomer)**			
[apo]	26 166	26 166	0
[2Fe-2S]^2+^	26 340	26 339	−1
[4Fe–4S]^2+^	26 516	26 515	−1
**(ArnR)_2_**			
[apo]/[apo]	52 332	52 329	−3
[apo]/[4Fe–4S]^2+^(DEA)	52 755	52 757	+2
[apo]/[4Fe–4S]^2+^(DEA NONOate)	52 888	52 890	+2
[4Fe–4S]^2+^/[4Fe–4S]^2+^	53 032	53 032	0
***hmp*::(ArnR)_2_**			
*hmp::*[apo](S)/[apo](S)	70 770	70 770	0
*hmp*::[apo]/[4Fe–4S]^2+^	71 088	71 086	−2
*hmp*::[2Fe-2S]^2+^/[2Fe-2S]^2+^	71 086	71 086	0
*hmp*::[4Fe–4S]^2+^/[4Fe–4S]^2+^	71 438	71 438	0
**Nitrosylated (ArnR)_2_**			
*hmp*::[4Fe–4S]^2+^(NO)/[4Fe–4S]^2+^(NO)	71 468	71 468	0
[4Fe–4S]^2+^/[4Fe–4S]^2+^(NO)	53 062	52 062	0
[4Fe–4S]^2+^(NO)/[4Fe–4S]^2+^(NO)	53 092	53 091	−1
[4Fe–4S]^2+^(NO)_2_/[4Fe–4S]^2+^(NO)	53 122	53 120	−2

a The predicted mass depends on the cluster/cluster fragment charge because binding is assumed to be charge compensated. Here, the cluster is assumed to be in the +2 state.

b The observed mass is derived from at least two independent experiments, with standard deviation of ±1 Da.

c The difference between the observed and predicted masses.

### Analysis of ArnR-DNA complexes

**Figure 4.**
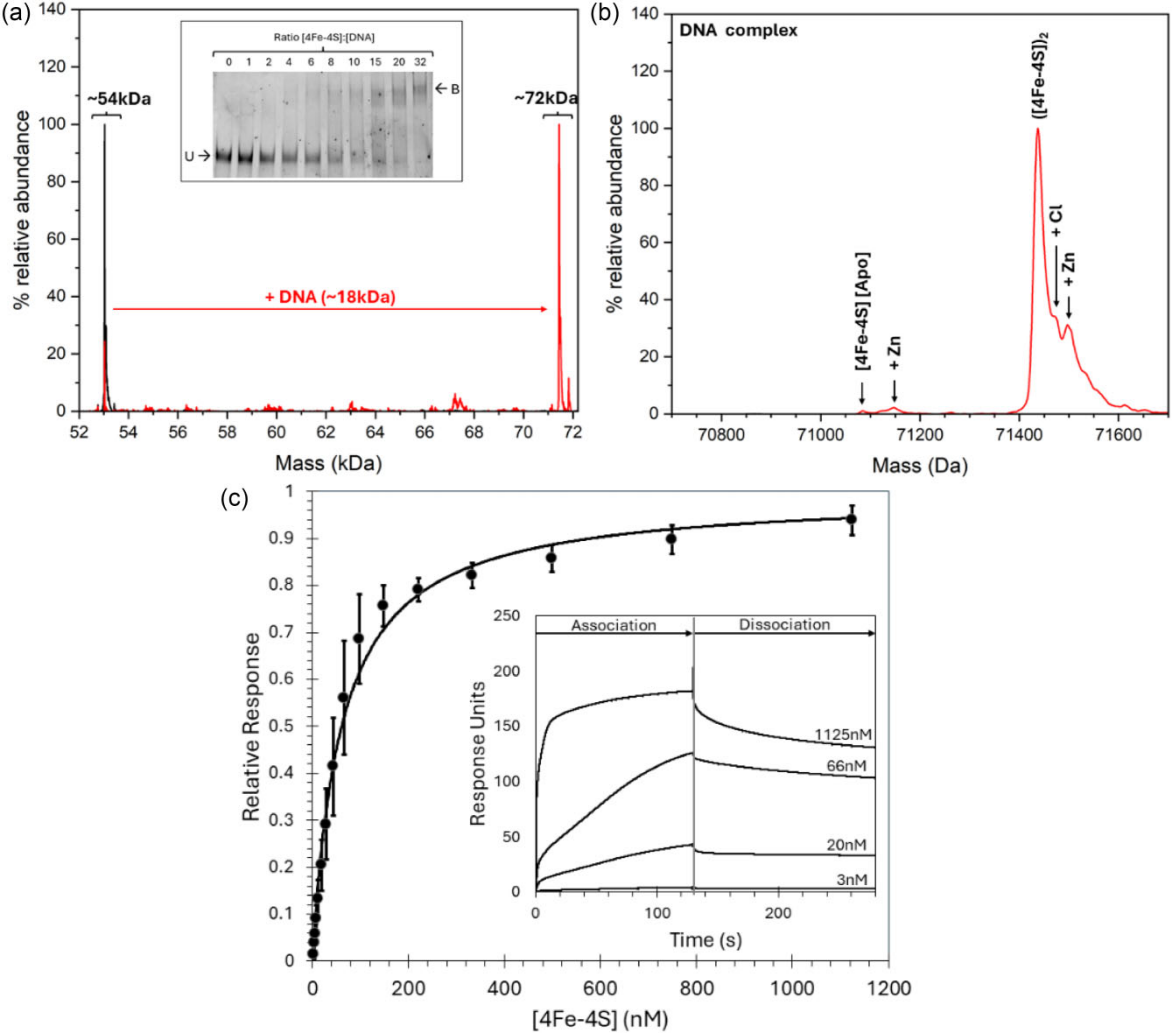
Analysis of ArnR-DNA complexes. (a) Deconvoluted mass spectrum of dimeric [4Fe–4S] ArnR before and after the stoichiometric addition of a 30 bp *hmp* promotor fragment, which led to protein-DNA complexes (as indicated). Inset is an EMSA of ArnR binding to an 80 bp *hmp* promotor. The binding buffer contained 10 mM Tris, 54 mM KCl 0.3% (v/v) glycerol, pH 7.5. (b) Deconvolution of protein-DNA complex over a narrower range indicated a requirement for two [4Fe–4S] clusters per dimer. (c) Analyte binding response of [4Fe–4S] ArnR to *hmp* promoter probed by SPR. A fit of the data to a simple one site binding equation is indicated by a solid line. Inset shows association and dissociation phases for selected concentrations of cluster.

*Nishimura et al*. identified the ArnR binding site in the promoter region of various ArnR regulated genes (e.g. *hmp*), and demonstrated a specificity for the Fe–S form of ArnR [12, 18]. Thus, it was of interest to investigate the binding properties of [4Fe–4S] ArnR to the *hmp* promoter. Electrophoretic mobility shift assay (EMSA) experiments were conducted with fluorescently (6-carboxyfluorescein) labelled oligonucleotides containing the previously identified binding site upstream of the *hmp* gene. The EMSA data for binding were sub-optimal, probably because of the probe size (80 bp), but they clearly showed some evidence for DNA binding as the concentration of [4Fe–4S] ArnR was increased, with maximal binding occurring at ∼315 nM [4Fe–4S] ArnR ([4Fe–4S]: [DNA] = ∼32), consistent with the observations of Nishimura *et al*. [12, 18] (Fig. 4a, inset).

The ability of native MS to resolve multiples species and the complexes they form could provide new insights not available from EMSA experiments [65]. For native MS, as isolated [4Fe–4S] ArnR was mixed ([4Fe–4S]: [DNA] = ∼1) with a small (30 bp) unlabelled double stranded oligonucleotide containing the *hmp* binding site. This led to the appearance of a new peak centred on 71.4 kDa that corresponded to the DNA-bound form of dimeric [4Fe–4S] ArnR, along with some unbound dimeric ArnR (Fig. 4a, see Table 1 for a comparison between observed and predicted masses).The region of the spectrum corresponding to the protein-DNA complex was dominated by a peak at 71 438 Da, indicative of the presence of DNA-bound dimeric ArnR containing two [4Fe–4S] clusters, together with +36 Da (Cl) and +63 Da (Zn) adducts to the high mass side. No evidence for apo ArnR binding to DNA was observed, but this was present in the sample at very low abundance (Fig. 4b).

Although EMSAs are often used to assess protein-DNA binding, both qualitatively and quantitatively, there are inherent disadvantages with EMSAs that can make quantitative characterization unreliable [66, 67], particularly when studying Fe–S proteins [45]. To gain a more detailed insight into the affinity of ArnR for the *hmp* promoter, SPR was utilized. SPR enables high sensitivity measurements of analyte binding ([4Fe–4S] ArnR) to an immobilized ligand, (*hmp* promoter DNA), yielding binding affinities, while overcoming many of the problems associated with determining DNA-binding affinities by EMSAs [66–69].

Here, we utilized the ReDCaT principle of SPR to obtain the binding affinity of ArnR for the *hmp* promoter [45, 46]. Binding was specific, since negligible binding of ArnR to the reference surface containing just the immobilized ReDCaT probe was detected. Satisfactory fits to the data could be obtained using a simple binding equation (see Equation 1), giving a *K*_d_ of 30 ± 3 nM for binding of the ArnR dimer to the *hmp* promoter (or 60 nM in terms of [4Fe–4S] cluster, Fig. 4c). Although a kinetic characterization of DNA binding was beyond the scope of this work, qualitatively, the association phase of the SPR sensorgram indicated rapid ArnR binding to the *hmp* sequence, with a slow dissociation phase, consistent with relative stability of the ArnR-DNA complex in the absence of NO (Fig. 4c, inset), as suggested previously [12, 18].

### ArnR is not an O_2_ sensor

*In vivo*, ArnR is constitutively expressed irrespective of the presence of O_2_ [11, 12, 18, 19]. The aerobic repression of the *narKGHJI* operon by ArnR contrasts with the anaerobic activation of the equivalent *E. coli* operon by [4Fe–4S] FNR [37]. We note that Nishimura *et al*. have previously reported that Fe–S ArnR exhibits a degree of sensitivity towards O_2_ and NO [12, 18]. To learn more about these processes, we investigated the response of as-isolated [4Fe–4S] ArnR towards O_2_ and NO (see below).

To investigate the O_2_ reactivity, [4Fe–4S] ArnR was diluted into aerobic buffer (22 µM in cluster) containing dissolved atmospheric O_2_ (∼220 µM) and spectra recorded over time (Fig. S4a). The [4Fe–4S] cluster exhibited remarkable stability to O_2_, consistent with its role in the aerobic repression of the *C. glutamicum narKGHJI* operon (Fig. S4a, inset). Over a period of hours, the [4Fe–4S] cluster eventually succumbed to O_2_, leaving apo protein. These observations are consistent with those of Nishimura *et al*. The low O_2_-reactivity of ArnR in the presence of promoter DNA was also probed using native MS. The AnrR-DNA complex was still present after ∼3 hr following exposure to O_2_. Intermediates of any cluster conversion process were not readily detected (Fig. S4b–d), with only the most stable species detected, as previously observed for the O_2_-tolerant, but NO-sensitive, L28H variant of FNR [70]. Oxygen and (likely) sulfur adducts of apo ArnR and the [2Fe–2S] cluster degradation intermediate were observed.

Determining the primary analyte of a response regulator can be difficult, especially where limited *in vivo* or *in vitro* studies exist [71]. For ArnR, there is sufficient *in vivo* and *in vitro* evidence to suggest that ArnR senses and responds to endogenous NO [11, 12, 18, 28]. Thus, [4Fe–4S] ArnR is unlikely to function as a primary O_2_ sensor but could, conceivably, fulfil a secondary O_2_-sensing role [28, 72].

### ArnR is a NO sensor

**Figure 5.**
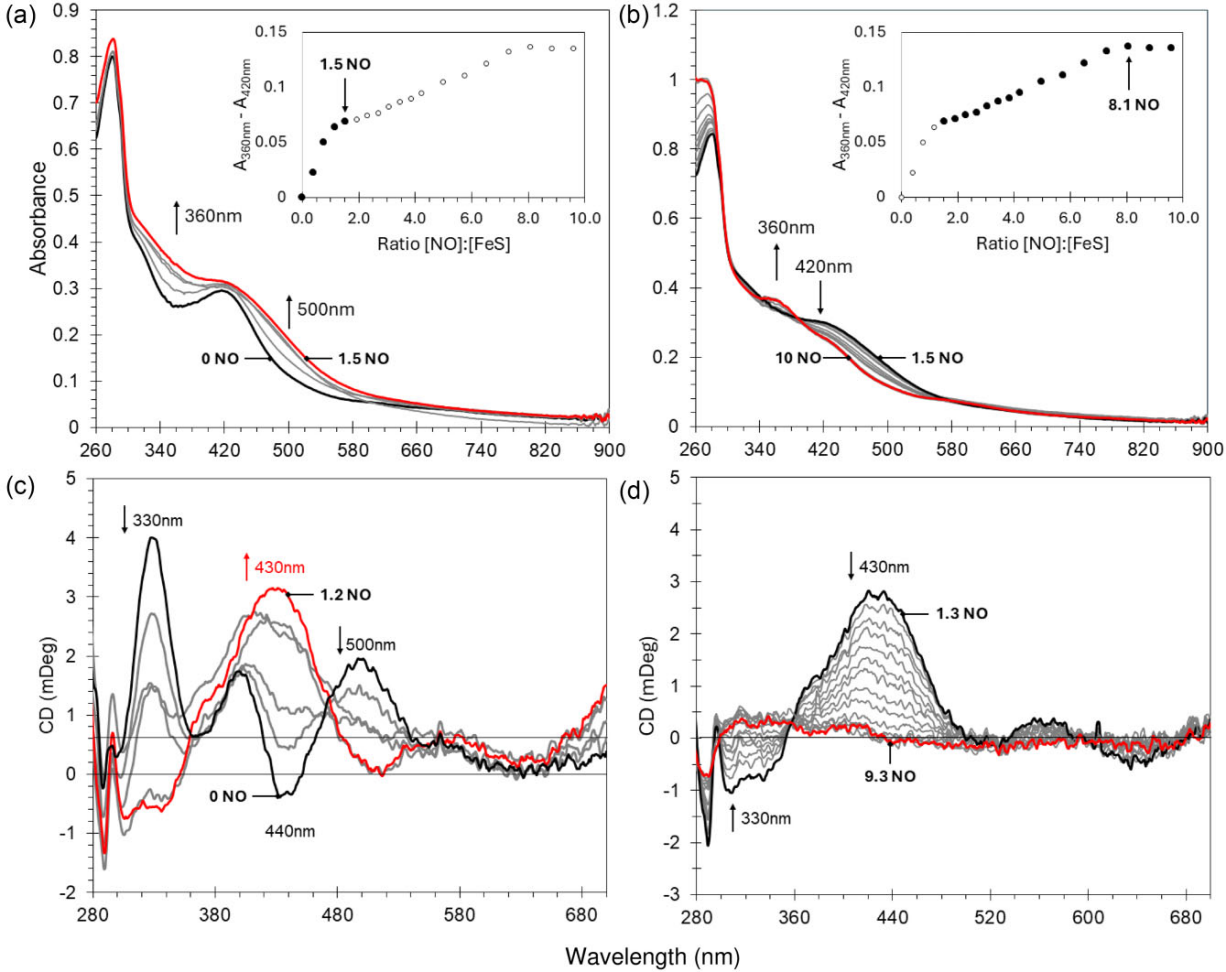
Titrations of [4Fe–4S] ArnR with NO. Absorption spectra of [4Fe–4S] ArnR following sequential additions of NO, where (a) [NO]: [Fe–S] 0–1.5 and (b) 1.5–10, as indicated. Inset shows absorbance changes (A360–A420 nm) as a function of [NO]: [Fe–S]. Circular dichroism spectra of an equivalent sample where (c) [NO]: [Fe–S] 0–1.2 and (d) 1.3–9.3, as indicated. [NO]: [Fe–S] intervals are ∼0.25 and ∼0.44 for (c) and (d), respectively. Changes in CD signal at 300, 400, 440, and 500 nm as a function of [NO]: [Fe–S] are shown in Fig. S5.

The reaction of [4Fe–4S] ArnR with NO was investigated by measuring changes in the cluster absorption following sequential additions of NO under anaerobic conditions. Initial increases in intensity were observed across the 300–600 nm region, with a broad isosbestic point at ∼420 nm (Fig. 5a). As the titration progressed, further changes occurred, with simultaneous increase in A360 nm and decrease in A420 nm. The final spectrum, with a principal absorption at 360 nm and minor shoulder at 430 nm, was consistent with the formation of iron-nitrosyl species, and closely resembles the spectra of products formed upon nitrosylation of *Streptomyces coelicolor* NsrR and other NO-sensitive Fe–S proteins (Fig. 5b) [73–75]. These were initially assigned to Roussin’s red ester (RRE)-like species, which exhibit a principal absorption band at 362 nm and a shoulder at 430 nm, but a more complex picture of nitrosylation, involving multiple species, has now emerged [76–79]. A plot of A360–A420 nm versus [NO]: [4Fe–4S] contained a clear break point at a stoichiometry of 1–2 NO per cluster (Fig. 5a, inset), with the reaction essentially complete at a stoichiometry of ≥8 NO molecules (Fig. 5b, inset), observations that are reminiscent of *S. coelicolor* NsrR [75] and *E. coli* L28H FNR [70].

CD spectroscopy was used to follow the reaction with NO. Sequential NO additions resulted in major changes to the CD band pattern. The starting spectrum contained three positive bands at (+) 330, 400, and 500 nm, together with a major negative band at (−) 440 nm. As NO was added ([NO]: [4Fe–4S] ≤ 2), the intensity of the (+) 330 nm band decreased significantly, becoming negative and splitting into two bands at (−) 306 nm and (−)335 nm. The (+) 400 nm, and (−) 440 nm bands shifted, becoming a single broad intense positive feature at (+) 430 nm, while the (+) 500 nm band was lost (Fig. 5c). As further NO was added, [NO]: [4Fe–4S] ≥ 2, no further band shifts were observed and the CD spectrum collapsed towards zero intensity, with little change after the addition of ∼7 NO (Fig. 5d). A plot of CD intensity at 330 or 500 nm showed that changes were complete at ∼1 NO per cluster, with only minor changes at higher [NO]: [4Fe–4S] ratios.

Equivalent plots of CD intensity changes at 400 and 440 nm clearly indicated the formation of an intermediary species, reaching a maximum between 1 and 2 NO per cluster, which subsequently reacted with further NO to give an achiral species at >8 NO (Fig. S5), reminiscent of NsrR observations [75]. The UV–visible absorbance and CD spectroscopy reported here clearly show the rapid formation of a potentially stable intermediate following 1–2 NO, as observed previously for NsrR [75].

### Nitrosylation of the cluster causes a loss of DNA binding

To learn more about the nature of the intermediary species detected by optical methods, additional biophysical methods are necessary. We have previously applied native MS to study the nitrosylation of [4Fe–4S] NsrR, which resulted in detection of mono- and di-nitrosylated [4Fe–4S] NsrR species, prior to the formation of protein-associated iron-nitrosyl species that are closely related to dinitrosyl iron complex {DNIC, [Fe(NO)_2_(L)_2_]}, Roussin's red ester {RRE, [Fe_2_(NO)_4_(L)_2_]} and Roussin's black salt-like {RBS-like, [Fe_4_S_3_(NO)_7_]} species, consistent with data from nuclear resonance vibrational resonance spectroscopy [78, 80, 81]. Similar observations have also been made for site-differentiated [4Fe–4S](L_3_)(X) model complexes [81, 82]. As these previous nitrosylation experiments were carried out in the absence of DNA, the physiological relevance of protein bound iron-nitrosyls remains unclear. Thus, the effect of NO on ArnR-DNA complexes was investigated using native MS.

**Figure 6.**
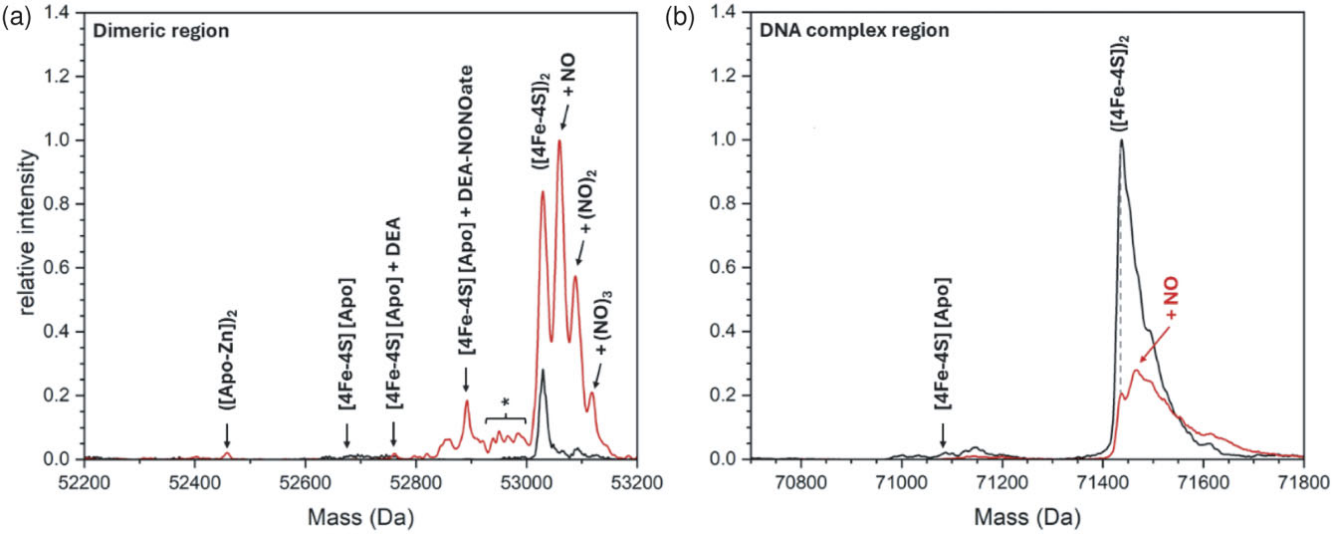
Nitrosylated [4Fe–4S] ArnR dimers no longer bind DNA. (a) Pre-formed [4Fe–4S] ArnR-DNA complexes before (lower intensity spectrum) and after (higher intensity spectrum) exposure to ∼2 NO per cluster were analysed by native MS in the dimeric mass region. (b) As in (a) but in the ArnR-DNA complex region, with the higher intensity spectrum corresponding to that before addition of NO. An asterisk indicates some clusters were damaged during ionization (e.g. [4Fe-3S]) [64, 90]. The increase in non-DNA-bound dimeric [4Fe–4S] ArnR observed following addition of NO, along with nitrosylated forms, most likely arises due to loss of NO during the measurement (i.e. dissociation from DNA resulted from nitrosylation).

Here, we used the *in situ* native MS nitrosylation methodology, as previously described for NsrR [80, 81, 83]. Holo ArnR samples were prepared in complex with *hmp* promoter DNA, then treated with the slow NO release agent DEA NONOate. Decomposition of the NONOate controls NO availability and limits the rate of reaction, enabling an effective thermodynamic titration [80, 84]. Spectra recorded in the absence of NONOate were dominated by a peak at 71 432 Da, consistent with the presence of DNA-bound [4Fe–4S] ArnR dimers, together with some uncomplexed [4Fe–4S] ArnR dimers (Fig. 6, black line). Following the addition of ∼2 [NO]: [4Fe–4S], there was a marked increase in the amount of uncomplexed ArnR present in the spectrum (Fig. 6a). In the region of the spectrum corresponding to the protein-DNA complex, a clear +30 Da adduct was observed for the holo AnrR *hmp* complex (Fig. 6b, red line, Fig. S6, and Table 1 for a comparison between observed and predicted masses). In the dimer region of the spectrum, clear evidence for ArnR dimers with 1–3 NO molecules per dimer was apparent, but there was little evidence for further iron-nitrosyl formation, (e.g. DNIC, RRE, and RBS), or of a significant increase in the abundance of apo ArnR dimers, or of S-nitrosylation (Fig. 6a, red line). These observations indicate that mono-nitrosylated ArnR dimers retain the ability to bind DNA to some extent, but are also found dissociated from DNA. The fact that we do not observe the formation of di-nitrosylated ArnR in complex with DNA suggests the binding of 1–2 NO molecules is sufficient to induce a loss of DNA binding.

By analogy to other transcriptional regulators, the requirement for ∼2 analyte molecules (in this case NO) per dimer is not unusual. For instance, dimers of the heme-dependent carbon monoxide (CO) oxidation activator (CooA), which belongs to the CRP/FNR transcriptional regulator superfamily family, become competent for DNA binding upon binding two molecules of CO, one per heme cofactor [85]. Similarly, dimeric cyclic-AMP (cAMP) receptor protein (CRP) becomes competent for DNA binding upon binding of two cAMP molecules [86–88]. The interaction of a single molecule of O_2_ with dimeric [4Fe–4S] FNR initiates cluster conversion and may be sufficient to promote monomerization, causing a loss of DNA binding [16, 89]. More recently, we have shown, via native MS, that mono-nitrosylation of the [4Fe–4S] NsrR dimer is sufficient to abolish DNA binding [83]. Hence, transcriptional regulators only require near stochiometric amounts of their analyte to elicit changes in DNA transcription, enhancing sensitivity to fluctuations in analyte concentration within the cytosol.

The concentration of endogenously produced NO in the bacterial cytosol during nitrate respiration has not been determined directly, but recent literature indicate it is likely to be sub-micromolar [91]. This has led to an ongoing disagreement within the literature concerning the likely physiological significance of *in vitro* nitrosylation experiments, where there is a potential risk of shifting from physiologically-relevant biochemistry, to interesting but likely *in vitro* chemistry [92, 93]. By studying the effect of NO on transcriptional regulator-DNA complexes, we now have a clearer picture of what part of the nitrosylation process is physiologically relevant and what is unlikely to be [80, 81]. Here, we have shown that mono-nitrosylation of each [4Fe–4S] cluster present in the ArnR dimer is sufficient to abolish DNA binding, with similar observations have recently been reported for NsrR [83]. It also indicates that Fe–S clusters do not have to undergo degradation in order to sense NO. Finally, this study highlights the power of native MS to provide unprecedented insight into the mechanisms of analyte-sensing transcriptional regulators.

## Discussion

The influence of nitrate and nitrite on the growth of *C. glutamicum* is well documented [11, 12, 18, 19, 94]. The operon encoding nitrate reductase (*narKGHIJ*) is controlled by at least two transcriptional regulators (GlxR and ArnR) and repressed under aerobic conditions [12, 18, 19]. GlxR, a member of the CRP/FNR superfamily, controls ∼100 different genes involved in a wide range of aspects of aerobic and anaerobic respiration, nitrogen assimilation, central metabolism, and stress response. GlxR activates the expression of *narKGHJI* in response to cAMP, a secondary messenger of energy status. In contrast, ArnR is a strong repressor of *narKGHJI* under aerobic conditions, allowing only basal levels of expression [19, 95]. Here, we have shown that ArnR utilizes a [4Fe–4S] cluster as a sensory cofactor. Although we propose that [4Fe–4S] ArnR is unlikely to function as a direct O_2_ sensor, it does exhibit a degree of sensitivity towards O_2_. Thus, O_2_-induced turnover of the [4Fe–4S] cluster *in vivo* may contribute to the basal level of nitrate reductase expression seen during aerobic growth [19].

The *narKGHJI* operon encodes nitrate reductase and catalyses the reduction of nitrate (NO_3_^−^) to nitrite (NO_2_^−^). Where studied, nitrate reductases are also able to reduce NO_2_^−^ to NO when intracellular concentrations of NO_2_^−^ become elevated [21, 96]. In the presence of abundant intracellular O_2_, NO will be rapidly consumed by a variety of chemical processes that ultimately result in its removal [25, 97], limiting detection by ArnR. As intracellular O_2_ concentrations become limiting, this endogenously-produced NO will persist in the cytosol, increasing the likelihood of detection by [4Fe–4S] ArnR [98]. We have shown here that [4Fe–4S] ArnR is acutely sensitive to NO, forming a distinct chiral species following the addition 1–2 NO per cluster, and prior to the formation of an achiral species with absorbance properties reminiscent of RRE and/or RBS at ∼8 NO [70, 75, 77, 78, 80, 81, 99]. Our native MS observations provide conclusive evidence for the formation of mono- and di-nitrosyl ArnR species following the addition of ∼2 NO per cluster. Surprisingly, mono-nitrosylated ArnR dimers retained some ability to bind to the *hmp* promoter DNA, but the di-nitrosyl species did not. By analogy with other transcriptional regulators, we propose that this di-nitrosyl ArnR species contains two [4Fe–4S](NO) clusters per dimer and that its formation abolishes DNA binding [85]. Although the fourth ligand to the ArnR cluster not known, it seems likely that NO-induced dissociation of a differentiated ligand from the [4Fe–4S] cluster would result in changes within the sensory domain that are conveyed to the DNA-binding domain, as proposed for NsrR [16, 81, 100] and FNR [101–103].

**Figure 7.**
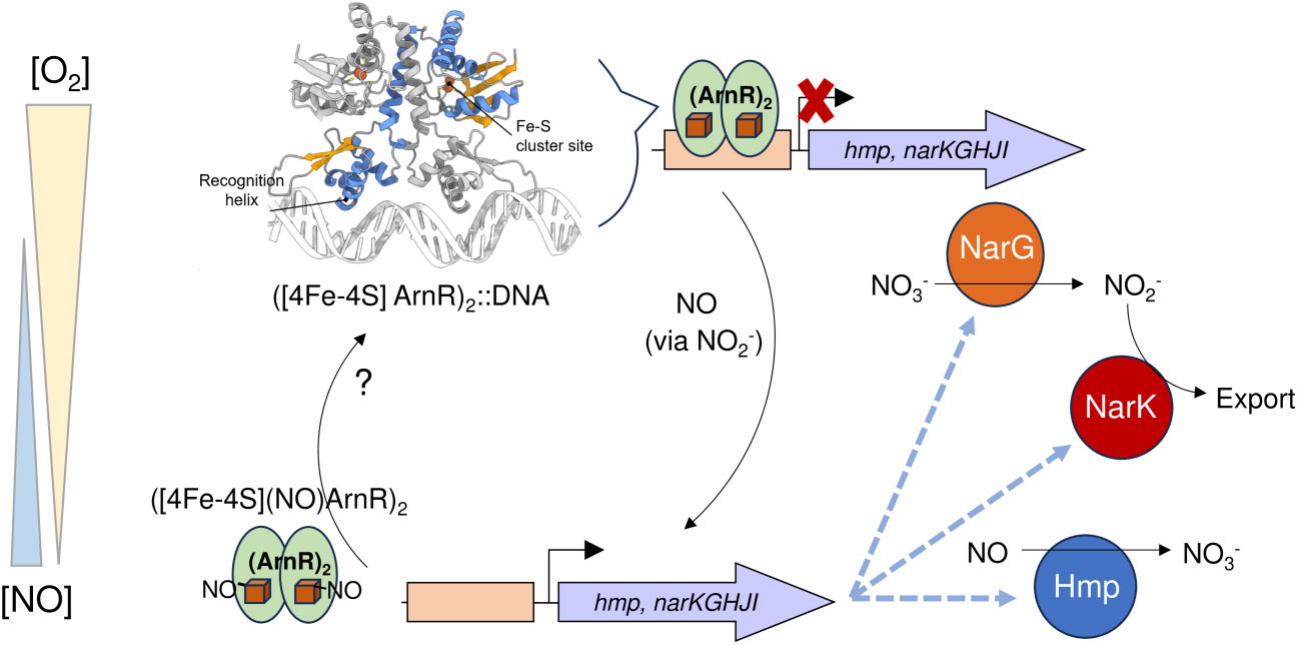
Proposed role of *C. glutamicum* anrr in the management of endogenous nitrosative stress. Basal levels of nitrate reductase (NarGHI) increase the cytoplasmic concentration of nitrite (NO_2_^−^). Accumulated nitrite competes for the active site of nitrate reductase (NarG), impairing nitrate (NO_3_^−^) reduction and resulting in the reduction of NO_2_^−^ to NO. Under aerobic conditions NO is rapidly eliminated, preventing accumulation and subsequent detection by arnr. Here, dimeric [4Fe–4S] arnr represses the transcription of *narKGHJI* and *hmp*, which encodes the NO-dexotifying enzyme hmp. At lower O_2_ tensions endogenously produced NO persists in the cytosol, increasing the likelihood of detection by anrr. Mono-nitrosylation of each [4Fe–4S] cluster in dimeric arnr relieves [4Fe–4S] arnr-mediated repression. expression of hmp lowers the cytoplasmic NO burden by recycling it to NO_3_^−^ when sufficient O_2_ is present, and possibly to N_2_O when it is not [94]. In addition, elevated *narKGHIJ* expression balances nitrate utilization (via narg) and nitrite export (via nark). The question mark indicates that the reversibility of nitrosylation/DNA binding is currently not known. Hence, NO is used as a proxy for nitrite to help optimize nitrate respiratory growth, as previously proposed for *E. coli* [23]. For clarity, the activating effect of glxr on *nar* expression [19] is not included in the scheme.

The synergistic loss of repression by ArnR and concomitant activation by GlxR would ensure timely expression of *narKGHJI* in response to declining O_2_ concentrations. Based upon the coordinate regulation of *narKGHJI* and *hmp* expression by the NO-sensitive repressor ArnR, we propose that endogenous NO, initially produced by basal levels of nitrate reductase (NarGHI) prevents the intracellular accumulation of both nitrite (via NarK) and NO in the cytosol (via Hmp) (Fig. 7). We note that *C. glutamicum* Δ*hmp* strains display strong growth defects under aerobic conditions in the presence of nitrate or nitrite, while under anaerobic conditions, the effect is less clear [18, 94], probably reflecting the need for O_2_ for efficient detoxification/conversion of NO. Furthermore, Δ*arnR* strains, which constitutively express Hmp, appear better able to cope with nitrate/nitrite metabolism [28, 94].

In summary, the regulator ArnR, which controls the switch to anaerobic respiration in *C. glutamicum*, utilizes a [4Fe–4S] cluster that does not respond directly to O_2_. Instead, it responds specifically to the presence of NO that accumulates when O_2_ levels drop. Reaction with NO results in the formation of a di-nitrosylated form of the [4Fe–4S] ArnR dimer, likely containing two [4Fe–4S](NO) species per dimer, which can no longer bind to promoter DNA. The observations presented here are consistent with previous *in vitro* and *in vivo* observations and help to clarify our understanding of how this novel transcriptional regulator functions [12, 18].

## Supplementary Material

mfaf026_Supplemental_File

## Data Availability

Data supporting the conclusions of this study are available in the main paper with additional experimental data given in the ESI. All data are available from the corresponding author upon request.
