# Supplementary material for: ArnR binds a [4Fe–4S] cluster and indirectly senses anaerobicity in Corynebacteria
Source: Metallomics. 2025 Jul 21;17(8):mfaf026. doi: 10.1093/mtomcs/mfaf026 (PMC12343034; doi:10.1093/mtomcs/mfaf026)
Supplement: mfaf026_Supplemental_File [file mfaf026_supplemental_file.docx]

**Supplementary data**

**ArnR binds a [4Fe-4S] cluster and indirectly senses anaerobicity in *Corynebacteria***

Jason C. Crack, Lauren R. Harvey, Katie E. Johnson, and Nick E. Le Brun

**Supplementary tables**

**Table S1.** Oligonucleotides using in this work.

| **Name** | **Sequence (5’ 🡪 3’)^a^** | **Use*^b^*** |
| --- | --- | --- |
| JC01_F | FAM//CGCCTTTCCATGCTTCATGGGTTAATTTCTACGGATT**T**  **AATCTAATTAAATAAATCC**CAGGATCCAAGCAATTTGGCCTT | EMSA |
| JC02_R | AAGGCCAAATTGCTTGGATCCTG**GGATTTATTTAATTAGATT**  **A**AATCCGTAGAAATTAACCCATGAAGCATGGAAAGGCG | EMSA |
| JC03_F | GGATT**TAATCTAATTAAATAAATCC**CAGGA | SPR |
| JC04_R | TCCTG**GGATTTATTTAATTAGATTA**AATCCCCTACCCTACGT  CCTCCTGC | SPR |
| JC05_F | GGATT**TAATCTAATTAAATAAATC**CCAGGA | nMS |
| JC06_R | TCCTGG**GATTTATTTAATTAGATTA**AATCC | nMS |
| ReDCaT_F | BIOTIN//GCAGGAGGACGTAGGGTAGG | SPR |
| ReDCaT_R | CCTACCCTACGTCCTCCTGC | SPR |

^a^Sequences are based on the *hmp* promoter from *C. glutamicum* ATCC 13032. The previously identified ArnR binding site is shown in bold [1, 2]. ^b^ ESMA, electrophoretic shift mobility assay. nMS, native mass spectrometry. SPR, surface plasmon resonance.

**Supplementary figures**


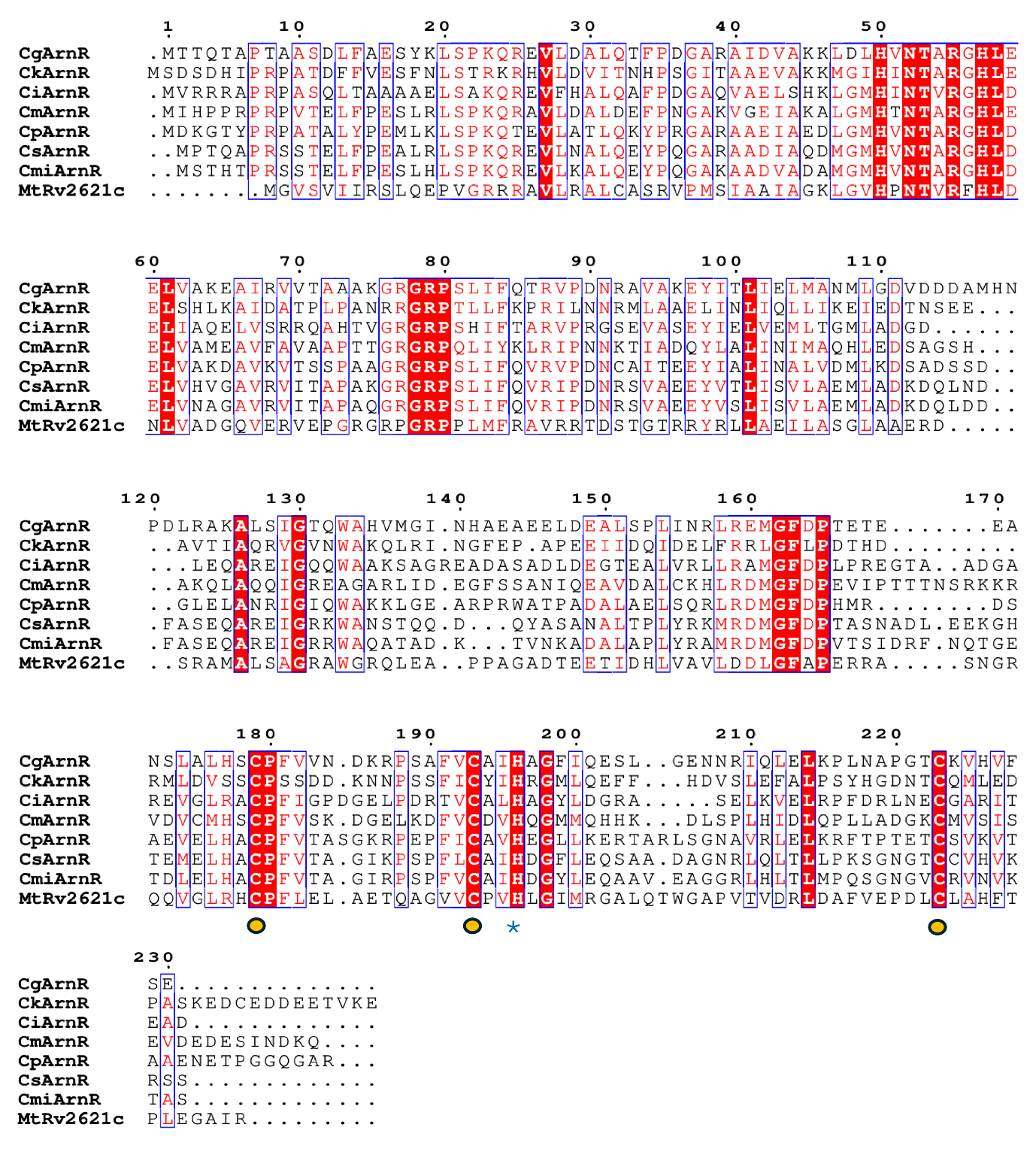


**Figure S1. Sequence alignment of ArnR homologs.** Alignment of *Corynebacterium glutamicum* strain ATCC 13032 ArnR (CgArnR, Cgl1185, UniProt ID Q8NR72), *C. kutscheri* ArnR (CkArnR, ID A0AB38VTT4), *C. imitans* ArnR (CiArnR, ID A0A239Y3Y0), *C. matruchotii* ATCC 14266 ArnR (CmArnR, ID A0A448TF55), *C. pilosum* ArnR (CpArnR, ID A0A376CPR9), *C. striatum* ArnR (CsArnR, ID A0AAQ1Z7C1), *C. minutissimum* ArnR (CmiArnR, ID A0A376CXR6) and *Mycobacterium tuberculosis* strain ATCC 25618/H37Rv Rv2621c (MtRv2621c, ID I6Y187). Sequences aligned in Clustal Omega [3] and visualised in ESPript 3.0 [4].

**
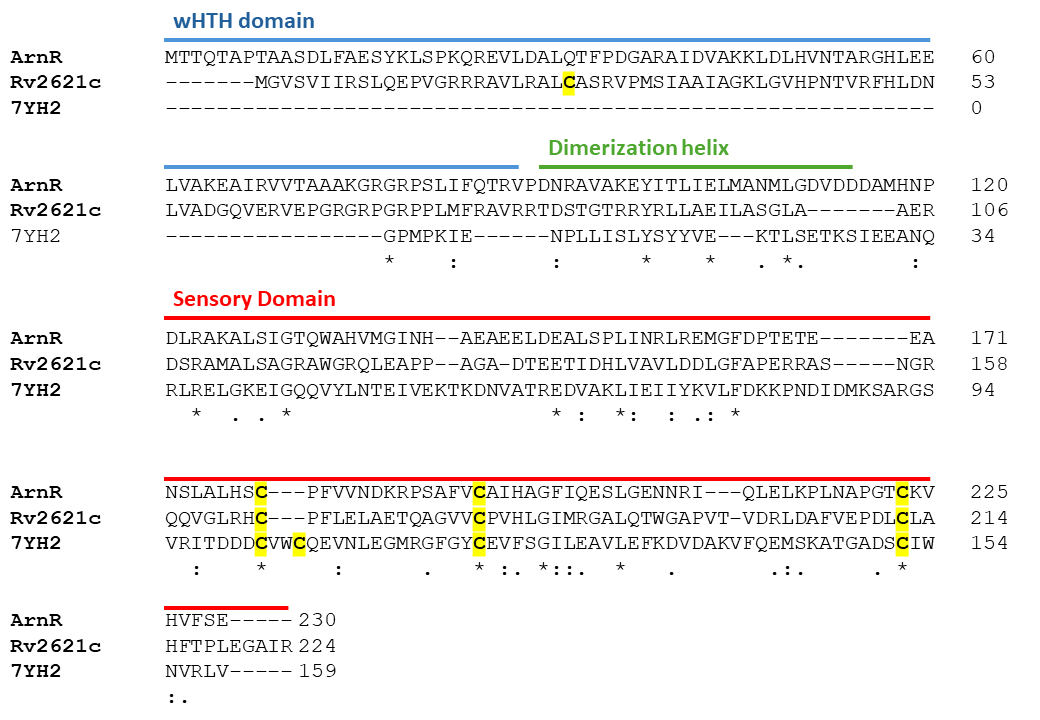
**

**Figure S2.** Clustal Omega alignment of ArnR with Rv2621c identified by Fold Seek and 7YH2 (TRAPPC3). Both ArnR and Rv2621c feature an ArsR-like winged helix-turn-helix DNA-binding domain (blue line), dimerization helix (green line) and sensory domain (red line). Rv2621c shares ~20% identity with ArnR. In addition, the sensory domain shares weak homology with the diverse TRAPPC3 family of proteins (PDB: 7YH2, CATH 3.30.1380.20). Cysteine residues are highlighted yellow. Conserved residues are marked with an asterisk (*); conservative substitutions with strongly similar properties are marked with a colon (:); substitutions resulting in weakly similar propertied are marked with a period (.); a hyphen (-) indicates sequence extension [3].


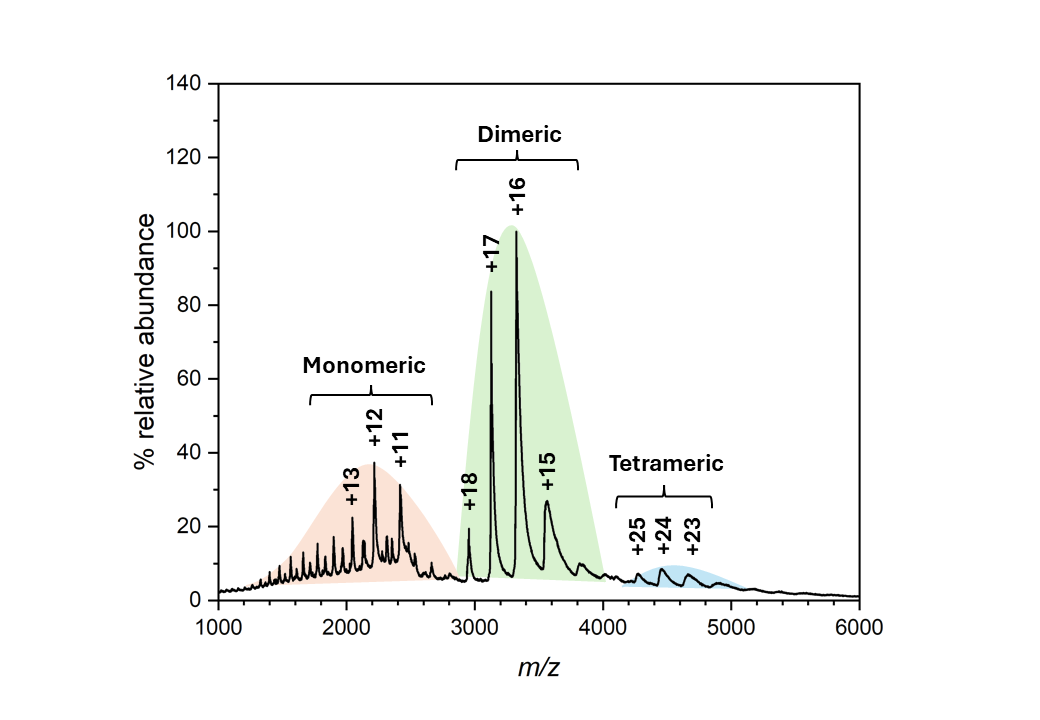


**Figure S3.** *m/z* spectrum of as-isolated ArnR. Speciation and charge numbers are annotated.


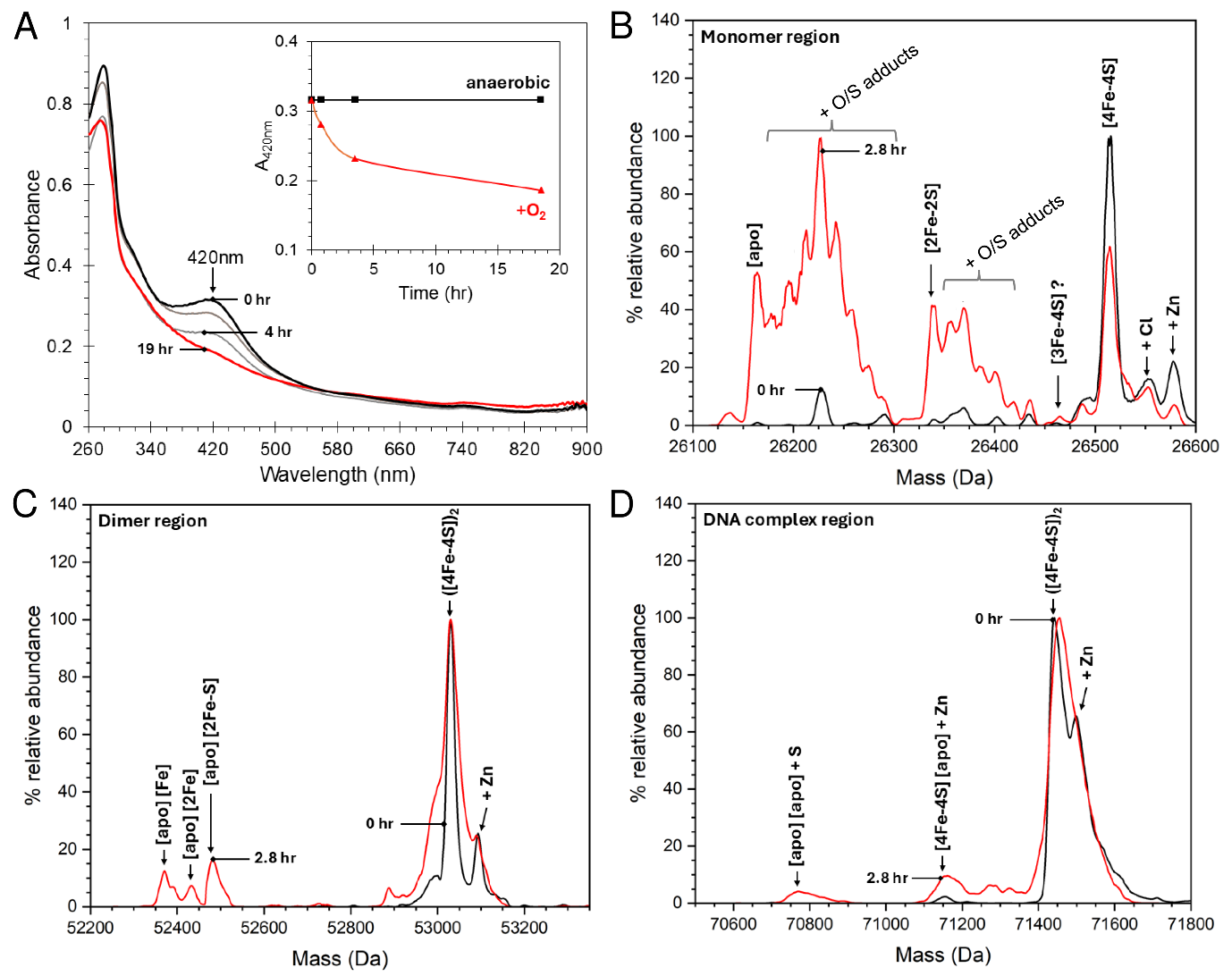


**Figure S4**. **[4Fe-4S] ArnR reacts slowly with O_2_.** (**A**) Absorbance spectra of [4Fe-4S] ArnR before and after exposure to O_2_ as a function of time; 0 hr (black line), 1 hr, 4 hr (grey lines) and 19 hr (red line) post exposure. Inset, plot of A_420 nm_ as a function of time post O_2_ exposure (red line) and anaerobic control (black line). (**B** – **D**) Native MS of ArnR in the (**B**) monomeric (**C**) dimeric and (**D**) DNA-complexed regions before (black line) and after (red line) ~3 hours exposure to O_2_.


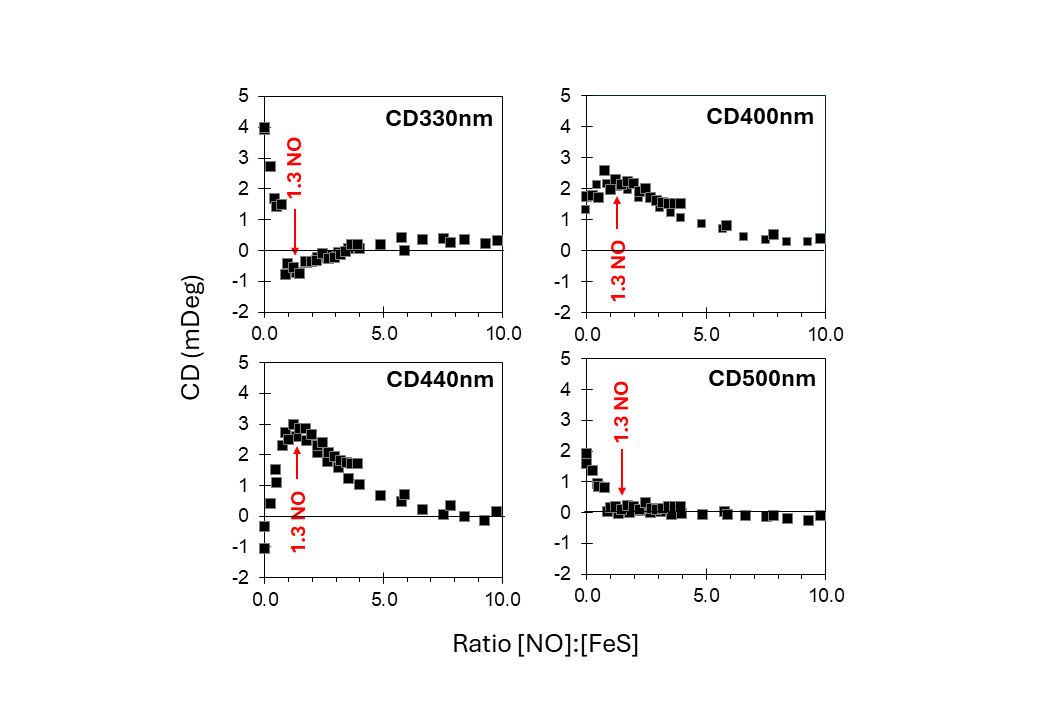


**Figure S5. Plots of CD intensity changes in the spectrum of ArnR as a function of NO concentration.** Changes at 330 nm, 400 nm, 440 nm and 500 nm, as indicated. A spectroscopically distinct intermediate was formed following the addition of 1 – 2 NO per cluster. Data points from two independent titrations are shown.


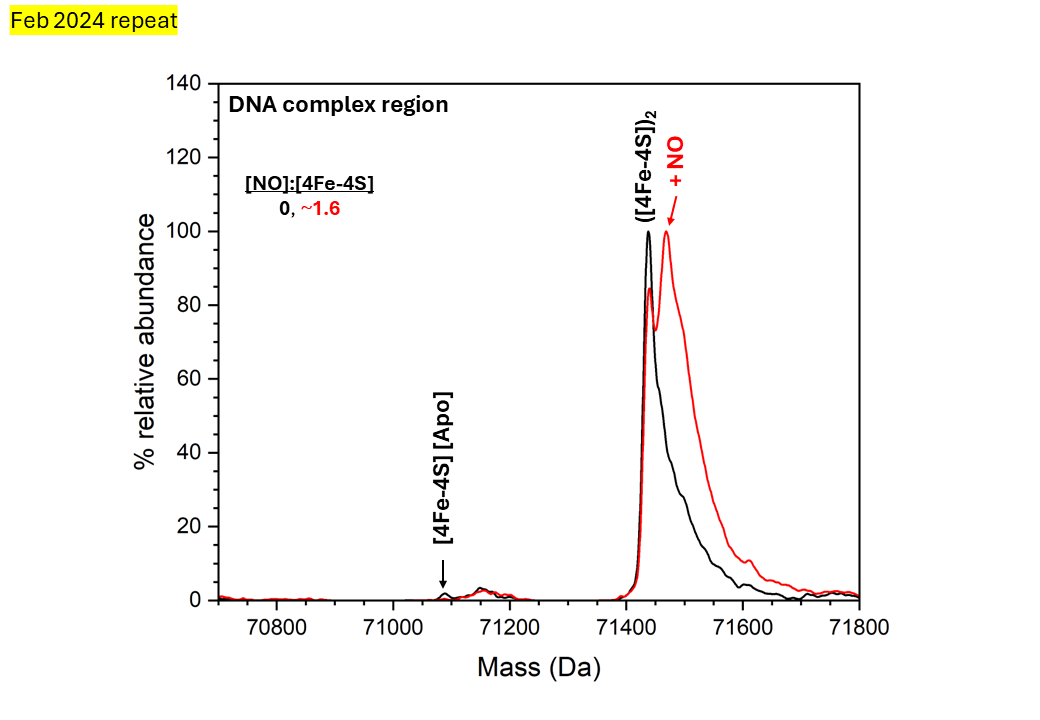


**Figure S6.** **Comparison of ArnR-DNA complexes in detail.** ArnR-DNA complexes pre (black line) and post (red line) NO exposure. See main text for details.

**Supplementary references**

1. Nishimura T, Teramoto H, Inui M *et al*. *Corynebacterium glutamicum* ArnR controls expression of nitrate reductase operon *narKGHJI* and nitric oxide (NO)-detoxifying enzyme gene *hmp* in an NO-responsive manner. *J Bacteriol* 2014;**196**:60-9. doi: 10.1128/JB.01004-13

2. Nishimura T, Teramoto H, Vertes AA *et al*. ArnR, a novel transcriptional regulator, represses expression of the *narKGHJI* operon in *Corynebacterium glutamicum*. *J Bacteriol* 2008;**190**:3264-73. doi: 10.1128/JB.01801-07

3. Sievers F, Wilm A, Dineen D *et al*. Fast, scalable generation of high-quality protein multiple sequence alignments using Clustal Omega. *Mol Syst Biol* 2011;**7**:539. doi: 10.1038/msb.2011.75

4. Robert X, Gouet P. Deciphering key features in protein structures with the new ENDscript server. *Nucl Acids Res* 2014;**42**:W320-4. doi: 10.1093/nar/gku316
